# Tonsillotomy

**Published:** 1902-06-14

**Authors:** 


### TONSILLOTOMY.

In the Ingleby lectures, delivered at the University of Birmingham, on Chronic Hypertrophy of the Faucial and Pharyngeal Lymphoid Tissue, Mr. F. Marsh spoke among other things of the operation of tonsillotomy. This operation he regards as necessary when the tonsils are so large that they mechanically cause obstructive symptoms; when they are the centres for frequent or prolonged catarrhal attacks or acute inflammatory attacks, when they are the cause of lymphadenitis, when the crypts or recesses are filled with caseating secretion which does not yield to treatment, and when they are the probable cause of a troublesome reflex cough. The operation of tonsillotomy in children is now seldom performed, he says, except under an anæsthetic, and in conjunction with an adenoid operation. But in older children it may be required alone, and in this case local anæsthesia by the application of a 10 per cent. solution of cocaine is all that is needed. If the child is nervous nitrous oxide gas may be used, and will give plenty of time. Mr. Marsh always uses Mackenzie's pattern of guillotine. The method of using it is all important. If the patient be sitting the head must be steadied by an assistant who must make steady pressure just beneath and behind the angle of the lower jaw at the time of the removal of the tonsil. The operator stands in front for the removal of the left tonsil, to the right and somewhat behind for the removal of the right one, *i.e.*, unless he is ambidextrous or the guillotine has a reversible handle. The guillotine should be warmed by dipping it in hot water.

The first step in the operation is the engagement of the tonsil, and here it is important to first encircle its lower edge. The second is to press the guillotine firmly outwards, and the third is to press the blade sharply home with the thumb. Inefficient removal of the tonsil is generally due to a non-recognition of the importance of the first two movements, to the use of too large a guillotine, or, more rarely, to adhesions between the tonsils and the pillars of the fauces preventing proper engagement of the tonsils within the ring of the guillotine. When the latter condition exists, it is sometimes necessary to separate the adhesions first. If the patient is under an anæsthetic a gag must be used, and it may be necessary to change it to the other side after the first tonsil is removed; this, however, is seldom required if the gag is carefully placed. In a combined operation, if the tonsils are so large as to interfere with the free exit of the curette or other instrument with the adenoids, they must be removed first, but if not it is better first to remove the adenoids. Removal of a superficial slice of tonsil is not enough, the greater part should be removed, only a small pad being left. After the operation no portion of tonsil should be seen projecting beyond the pillars of the fauces. Preferably there should be a slight hollow, but it is not desirable to enucleate the whole tonsil as may happen for example if the blade be not sharp by its slipping outside the tonsil.

Immediate hæmorrhage is sometimes free but generally stops of itself. Recurrent hæmorrhage is a more serious matter. The first step in its treatment is to ascertain whether it comes from a single vessel in one or both tonsils—in which case this should if possible be clamped with a Spencer Wells forceps—or from general oozing. If the latter the surface should be well scrubbed with adrenalin solution, and chloride of calcium, in doses of 5 to 10 grains according to age, should be given every hour until the bleeding stops. Or firm pressure may be made with a pad of lint, with or without adrenalin, which may be kept in position by the finger or by a pair of forceps. When there is suspicion of any hæmorrhagic tendency a preliminary dosing with calcium chloride is desirable. Secondary hæmorrhage is rare, but may occur as late as the sixth day from the opening up of a small vessel during the separation of a slough.

